# The PirB toxin protein from *Vibrio parahaemolyticus* induces apoptosis in hemocytes of *Penaeus vannamei*: PirB induces shrimp hemocytes apoptosis

**DOI:** 10.1080/21505594.2021.1872171

**Published:** 2021-01-25

**Authors:** Zhou Zheng, Ruiwei Li, Jude Juventus Aweya, Defu Yao, Fan Wang, Shengkang Li, Tran Ngoc Tuan, Yueling Zhang

**Affiliations:** a Department of Biology and Guangdong Provincial Key Laboratory of Marine Biotechnology, Shantou University, Shantou, China; b STU-UMT Joint Shellfish Research Laboratory, Shantou University, Shantou, China; c Southern Marine Science and Engineering Guangdong Laboratory, Guangzhou, China

**Keywords:** *Penaeus vannamei*, AHPND, PirB, hemocytes, nucleosome proteins, apoptosis, dephosphorylation

## Abstract

Acute hepatopancreatic necrosis disease (AHPND) is a major debilitating disease that causes massive shrimp death resulting in substantial economic losses in shrimp aquaculture. The Pir toxin proteins secreted by a unique strain of *Vibrio parahaemolyticus* play an essential role in the pathogenesis of AHPND. At present, most studies on the effects of Pir toxin proteins in shrimp focus on digestive tissues or organs such as hepatopancreas, stomach, etc., with none on the immune organs. In the present study, two recombinant Pir toxin proteins (rPirA and rPirB) of *V. parahaemolyticus* were expressed with rPirB shown to enter shrimp hemocytes. Employing pull-down and LC-MS/MS analysis, GST-rPirB was found to interact with 13 proteins in hemocytes, including histone H3 and histone H4 and among which histone H4 had the highest protein score. Further analysis using GST pull-down and Far-Western blot analysis revealed that rPirB could interact with histone H4. In addition, using the purified nucleosome protein from *Drosophila* S2 cells, it was found that PirB protein could specifically bind to histones. When flow cytometry was applied, it was observed that the interaction between PirB and histones in shrimp hemocytes induces apoptosis, which results in the dephosphorylation of Serine 10 in histone H3. Collectively, the current study shows that in addition to its effect on the digestive tract of shrimp, the PirB toxin protein interacts with histones to affect the phosphorylation of histone H3-S10, thereby inducing apoptosis.

## Introduction

The Pacific white shrimp, *Penaeus vannamei*, is one of the most important aquaculture shrimp species globally, accounting for nearly 80% or two-thirds of the total annual produce [1]. Unfortunately, the aquaculture industry suﬀers from various diseases and pathogenic infections caused by bacteria and viruses, which results in substantial economic losses [2,3]. Among shrimp bacterial diseases, acute hepatopancreatic necrosis disease (AHPND) is the most pathogenic and devastating disease that affects shrimp aquaculture [4]. Since the first outbreak of AHPND in China in 2009 and its subsequent spread to other countries such as Vietnam, Malaysia, Thailand, the Philippines, Mexico, etc., AHPND has caused billions of dollars in economic losses to farmers [5–7].

The causative agent of AHPND is a unique strain of *Vibrio parahaemolyticus* that contains a plasmid encoding the binary Photorhabdus insect-related (Pir) toxin genes PirA/PirB [8,9]. The AHPND-causing strain of *V. parahaemolyticus* (designated here as VP_AHPND_) contains a 70 kbp plasmid (designated pVA1) with sequence encoding for the PirA and PirB genes, which is absent in non AHPND-causing *V. parahaemolyticus* [9–13]. Removal of the pVA1 plasmid from VP_AHPND_ causes the bacteria to lose its ability to cause AHPND [11]. Similarly, selective knockdown of the Pir genes or removing the Pir toxins from VP_AHPND_ render the bacteria unable to cause AHPND in shrimp [11]. Even though PirA and PirB make up the binary toxin, PirB seems to be the main toxin protein that causes AHPND. For instance, shrimp did not display AHPHD symptoms when challenged with only PirA by reverse gavage, while the same treatment with only PirB resulted in typical symptoms of AHPND [11]. Similarly, when shrimp were cultured for 6 h in seawater containing a VP_AHPND_ isolate (designated 5HP), only PirB toxin was detected in the hepatopancreas of shrimp displaying the characteristic histopathological signs of AHPND, i.e. sloughing of the epithelial cells of hepatopancreatic tubules [14]. Recombinant PirB is more toxic to brine shrimp larvae compared with PirA [15]. The B subunit of PirAB^vp^ also recognizes glycosaminoglycan molecules (amino sugars) and participates in bacterial pathogenicity [16]. The PirA protein seems to play an auxiliary role in the pathogenesis of AHPND, while PirB alone could cause cellular damage, such as necrosis, apoptosis and other pathophysiological damage associated with AHPND [14].

Thus far, most of the AHPND associated cellular damage caused by PirB is found in the hepatopancreas, stomach and other digestive organs [14,17]. The effect of PirB on other shrimp tissues or organs is therefore worthy of evaluation, given that these toxins are transported to other tissues via hemolymph [14]. Moreover, a recent shrimp transcriptome study revealed the dysregulation of many genes, including apoptosis-related genes in hemocytes of VP_AHPND_-challenged *P. vannamei* [18]. The dysregulation of apoptosis-related genes in shrimp hemocytes suggests that VP_AHPND_ induces apoptosis in hemocytes, in addition to the typical AHPND-associated damage to hepatopancreas, stomach, and other digestive organs. Given that PirB is more toxic than PirA [15], the current study used various techniques to explore how the PirB toxin protein from *V. parahaemolyticus* induces apoptosis in shrimp hemocytes.

## Material and methods

### Experimental animals

*Penaeus vannamei* (weight of 8 ± 2 g each), bought from a local shrimp farm, Shantou Huaxun Aquatic Product Corporation (Shantou, Guangdong, China), were cultured in aerated laboratory tanks containing seawater (1% salinity) at 25°C for 3–5 days acclimatization to laboratory conditions before experiments. All animal experiments were performed according to the guidelines and approval of the Animal Research and Ethics Committees of Shantou University, Guangdong, China.

### Cloning, expression, and purification of recombinant PirA and PirB proteins and immunocytochemical analysis

The ability of the Pir toxin proteins to enter primary shrimp hemocytes was explored using immunocytochemical analysis. First, recombinant His-tagged PirA and PirB proteins (designated as His-rPirA or His-rPirB) were cloned, expressed, and purified as described previously [19]. Brieﬂy, the nucleotide sequence encoding PirA (GenBank #: KU145400.1) and PirB (GenBank #: KU145400.1) were ampliﬁed using the AHPND strain of *V. parahaemolyticus* (PD-2) (a kind gift from Dr. Chu Fang Lo of National Cheng Kung University) as template, with gene-specific primers (Table 1) designed with Primer Premier 5. The PCR product was cloned into pET-28a and transformed into *Escherichia coli* BL21 (DE3) (TransGen Biotech, Beijing, China). The expressed recombinant proteins were puriﬁed using Ni-NTA beads (GE Healthcare, CT, USA) according to the manufacturer’s instructions. The recombinant proteins were identified via sodium dodecyl sulfate-polyacrylamide gel electrophoresis (SDS-PAGE) and Western blot analysis as described previously [20]. The purified proteins (His-rPirA and His-rPirB) were separated on SDS-PAGE, before being transferred onto polyvinylidene ﬂuoride membranes (PVDF, Millipore, MA, USA). The PVDF membranes were blocked with 5% skimmed milk dissolved in TBST (20 mM Tris, 150 mM NaCl, 0.1% Tween 20, pH 7.4) for 1 h at room temperature. The membranes were incubated overnight at 4°C with mouse anti-His primary antibody (1:3000, Transgen BioTech, Beijing, China) followed by washing 3 times (15 min) with TBST. The membranes were then incubated for 1 h at room temperature with HRP-linked goat anti-mouse secondary antibody (1:3000, Sigma-Aldrich, MO, USA) and then washed 3 times (15 min). Protein bands on blots were detected with the Amersham Imager 600 system (GE Healthcare, CT).

**Table 1.** Sequence of primers used in this article

Primers	Sequence (5ʹ-3ʹ)
PirB-F-28a	agcaaatgggtcgcgTGACTAACGAATACGTTGTAAC
PirB-R-28a	cggagctcgaattcgCTACTTTTCTGTACCAAATTCA
PirB-F-GST	CCGGGATCCATGACTAACGAATACGT
PirB – R-GST	CGCCTCGAGCTACTTTTCTGTACCA
PirB-EGFP-F	CTTGGTACCGAGCTCGGATCCATGACTAACGAATACGT
PirB-EGFP-R	CCACACTGGACTAGTGGATCCCTTTTCTGTACCAAA
PirA-F	CCGGAATTCATGAGTAACAATATA
PirA-R	CCCAAGCTTTTAGTGGTAATAGATTGT
Histone H4-F	GGATCCATGACTGGACGTGGAAA
Histone H4-R	AAGCTTTTAACCTCCGAAACCGT

Next, immunocytochemical staining was used to analyze the Pir toxin proteins’ ability to enter primary shrimp hemocytes using a previously described method with minor modifications [21]. Briefly, hemolymph was withdrawn from 10 randomly selected shrimp with a sterile needle and syringe into an equal volume of precooled anti-coagulant buffer (27 mM C_6_H_5_O_7_Na_3_ · 2 H_2_O, 33 mM C_6_H_8_O_7_, 110 mM C_6_H_12_O_6_, 140 mM NaCl, pH 6.0). Samples were centrifuged immediately at 500 g for 10 min at 4°C to harvest the hemocytes, which were then resuspended in Insect-XPRESS™ (LONZA, Basel, Switzerland) media [22]. Next, 2 × 10^6^ hemocytes were seeded onto glass-bottom plates, incubated at 28°C for 2 h, before being treated with 0.1 µM His-rPirA or His-rPirB at 28°C for 2 h. Following this, cells were immediately fixed with 4% paraformaldehyde at room temperature for 15 min. Cells were washed three times with 0.01 M PBS (137 mM NaCl, 8 mM Na_2_HPO_4,_ 2 mM NaH_2_PO_4_, pH 7.4), followed by incubation with 0.5% Triton X-100 in 0.01 M PBS at room temperature for 20 min. Samples were then washed and incubated with 5% BSA (bovine serum albumin) for 30 min, and then with mouse anti-His antibody (TransGen BioTech, Beijing, China, 1:100) overnight at 4°C. After being washed, samples were incubated with goat anti-mouse IgG (H + L) Alexa Fluor 488 (Beyotime, Shanghai, China, 1:200) for 1 h at room temperature in the dark. Samples were washed three times, stained with Hochest 33,342 (Beyotime, Shanghai, China) for 10 min at room temperature protected from light, followed by washing six times, before being examined under a Confocal Microscope LSM800 (Carl ZEISS, Heidenheim, Germany).

### Pull-down assay

The proteins that potentially interact with the PirB protein in shrimp hemocytes were analyzed using pull-down experiments, as previously reported [23]. First, recombinant GST-tagged PirB protein (designated GST-rPirB) was cloned, expressed in the pEGX-6p-1 plasmid (TransGen Biotech, Beijing, China) before being puriﬁed using glutathione sepharose beads (GE Healthcare, CT, USA) according to the manufacturer’s instructions. Next, shrimp hemocytes harvested as described in subsection 2.2, were thoroughly washed three times with 0.01 M PBS before being lysed at 4°Cfor 30 min with lysis buffer (containing 25 mM HEPES, 150 mM NaCl, 1 mM EDTA-Na_2_ · 2H_2_O, 1% Triton X-100 and 1× Protease Inhibitor Cocktail Tablets, Roche, Switzerland), and then centrifuged at 20,000 g for 30 min at 4°C to collect the supernatant. Finally, lysates were incubated for 2 h at 4°C with glutathione sepharose beads (to which GST-rPirB or rGST has been coupled to) and then centrifuged at 1,000 g for 5 min at 4°C. After discarding the supernatant, samples were washed five times with 1% Triton X-100 in 0.01 M PBS, and then incubated with PreScission protease (GE Healthcare, CT, USA) for 16 h at 4°C, before being centrifuged at 1,000 g at 4°C for 2 min. Supernatants were collected and analyzed by SDS-PAGE.

### Protein identiﬁcation by mass spectrometry

The SDS-PAGE diﬀerential gel bands on the experimental samples from the pull-down assay were excised and submitted to a commercial company, Shanghai Applied Protein Technology (Shanghai, China), for LC-MS/MS analysis as described previously [24]. Briefly, the excised gel pieces were washed and de-stained at room temperature for 30 min. Samples were dehydrated and incubated with 10 mM DTT at 56°C for 30 min. Following this, samples were incubated with alkylation solution (10 mM IAA) at room temperature for 20 min, dehydrated followed by enzymatic digestion with sequencing grade modified trypsin (Promega Corporation, Madison, USA). Next, the samples were loaded onto a C18 nanoLC trap column and washed with Nano-RPLC Buffer A. An elution gradient of 5–40% acetonitrile (0.1% formic acid, 95% acetonitrile/water) in 60 mins gradient was used on an analytical ChromXP C18 column with spray tip. Data were acquired with a Triple TOF 5600 System (AB SCIEX, MA, USA), and the generated LC-MS/MS spectra searched against the NCBI database (http://www.ncbi.nlm.nih.gov/) using MASCOT (V2.3.02).

### GST pull-down analysis

The technique of GST pull-down was used to determine the interaction between PirB and histone H4, as described previously [25]. First, *P. vannamei* histone H4 (GenBank #: MH311299.1) was cloned with a His-tag and expressed in the pET-32a plasmid (TransGen Biotech, Beijing, China) as described in subsection 2.2. Next, beads coupled with rGST-PirB were incubated for 0.5 h at room temperature with supernatant extracted from the pET-32a-*Pv*histone H4 expressed in *E. coli* BL21. Samples were then centrifuged and washed. Finally, samples were analyzed by SDS-PAGE and Western blot, as described in subsection 2.2, with mouse anti-His primary antibody (1:3000, Transgen BioTech, Beijing, China).

### Far-Western blot analysis

Far-Western blot analysis, as previously described [20], was also used to ascertain the interaction between PirB and histone H4. First, 1 µg of the purified proteins (i.e. rGST, rGST-PirB or rPiB) were separated on SDS-PAGE and transferred onto PVDF membranes. Next, membranes were blocked with 5% skimmed milk dissolved in TBST before being incubated for 1 h at room temperature with the supernatant from the pET-32a-*Pv*histone H4 lysate extracted from *E. coli* BL21. Membranes were then incubated with mouse anti-His antibody (1:3000, Transgen BioTech, Beijing, China) followed by HRP-linked goat anti-mouse antibody (1:3000, Sigma-Aldrich, MO, USA) as described in subsection 2.2.

### Analysis of the interaction between PirB and nucleosome proteins

In the absence of shrimp cell lines, nucleosomes used to analyze nucleosome proteins’ interaction with PirB were purified from *Drosophila* Schneider 2 cells (a kind gift from Jianguo He of Sun Yat-sen University, Guangzhou, China). First, nuclear fractions were extracted as described previously with slight modifications [26]. Cultured S2 cells were collected, centrifuged, and washed twice with cold 0.01 M PBS. Cell pellets were resuspended in hypotonic buffer (10 mM Tris, 2 mM MgCl_2_, 0.5 mM PMSF, pH 7.5), incubated on ice for 5 min followed by the addition of 5.0 µL of 20% Triton-X-100 and the shearing of cells by passing through a 21 G needle with a 1 ml syringe at least 10 times. Next, samples were incubated on ice for 5 min before being centrifuged at 3,000 g for 4 min at 4°C to collect the pellet as nuclear fraction. After purifying the nuclear proteins, samples were centrifuged at 20,000 g for 20 min at 4°C and then incubated at 37°C with Micrococcal Nuclease (MNase, NEB, Beijing, China) for 90 min. The supernatant was collected for gel ﬁltration chromatography (Supersose™ 6 increase 10/300 GL, GE healthcare, CT, USA) on an AKTA pure 25 system (GE healthcare, CT, USA). Finally, all separated components were collected and analyzed using SDS-PAGE or Native-PAGE and Western blot, as described in subsection 2.2, with mouse anti-histone H3 antibody (1:1000, Transgen BioTech, Beijing, China). The obtained nucleosome proteins were used for subsequent experiments.

The interaction between PirB and nucleosome was analyzed as described previously with slight modiﬁcations [27]. Brieﬂy, purified proteins, i.e. His-rPirB and His-rPirA (negative control) were incubated at 37°C with puriﬁed nucleosomes for 30 min and then analyzed by Native-PAGE and Western blot as described in subsection 2.2, with mouse anti-histone H3 antibody (1:1000, Transgen BioTech, Beijing, China).

### Apoptosis detection using Annexin V-FITC/PI staining

To analyze whether PirB protein induces apoptosis in hemocytes, primary shrimp hemocytes were treated with rPirB. Briefly, hemocytes collected from shrimp hemolymph were cultured at 28°C for 2 h, before being treated for 4 h at 28°C with 0.1 µM rPirB, PBS (negative control), or apoptosis inducer A (positive control) (Beyotime, Shanghai, China). Next, cells were collected and stained with Annexin V-FITC/Propidium Iodide apoptosis detection kit (Beyotime biotechnology, Shanghai, China) followed by flow cytometry analysis using an Accuri C6 plus flow cytometer (BD Bioscience, SanDiego, USA) based on 10,000 events recorded on the dot plot.

To further ascertain the ability of PirB protein to induce apoptosis in shrimp hemocytes, *in vivo* experiments were carried out. First, healthy laboratory acclimatized shrimp were divided into two groups (30 each). One group of shrimps was each injected at the third abdominal segment with 0.8 µM rPirB, while the other group of shrimps was each injected with an equal volume of PBS (negative control). Hemocytes collected at 0, 12, 24, 36, and 48 h post-treatment were stained with the Annexin V-FITC/Propidium Iodide apoptosis detection kit and analyzed by flow cytometry. Triplicate samples were analyzed per treatment for at least three independent experiments.

### Detection of histone H3 phosphorylation in shrimp hemocytes

The phosphorylation of histone H3 by PirB was determined using Western blot analysis by treating primary shrimp hemocytes with rPirB. Briefly, hemocytes were collected and cultured, followed by treatment with different concentrations of His-rPirB or His-rPirA (0.2 µM or 0.8 µM) at 28°C for 2 h. Next, cells were lysed for 10 min at room temperature with lysis buffer (containing 25 mM HEPES, 150 mM NaCl, 1 mM EDTA-Na_2_ · 2H_2_O, 1% Triton X-100, 0.5% SDS and 1× Protease Inhibitor Cocktail Tablets, Roche, Switzerland) before being centrifuged at 20,000 g for 30 min at 15°C to collect the supernatant. The collected supernatant was analyzed using SDS-PAGE and Western blot, as described in subsection 2.2, with mouse anti-histone H3 antibody (1:1000, Transgen BioTech, Beijing, China), and rabbit anti-phospho histone H3 (Ser10) antibody (1:1000, Cell Signaling Technology, MA, USA).

## Results

### *The PirB toxin protein of* Vibrio parahaemolyticus *can localize to shrimp hemocytes*

**Figure 1.**
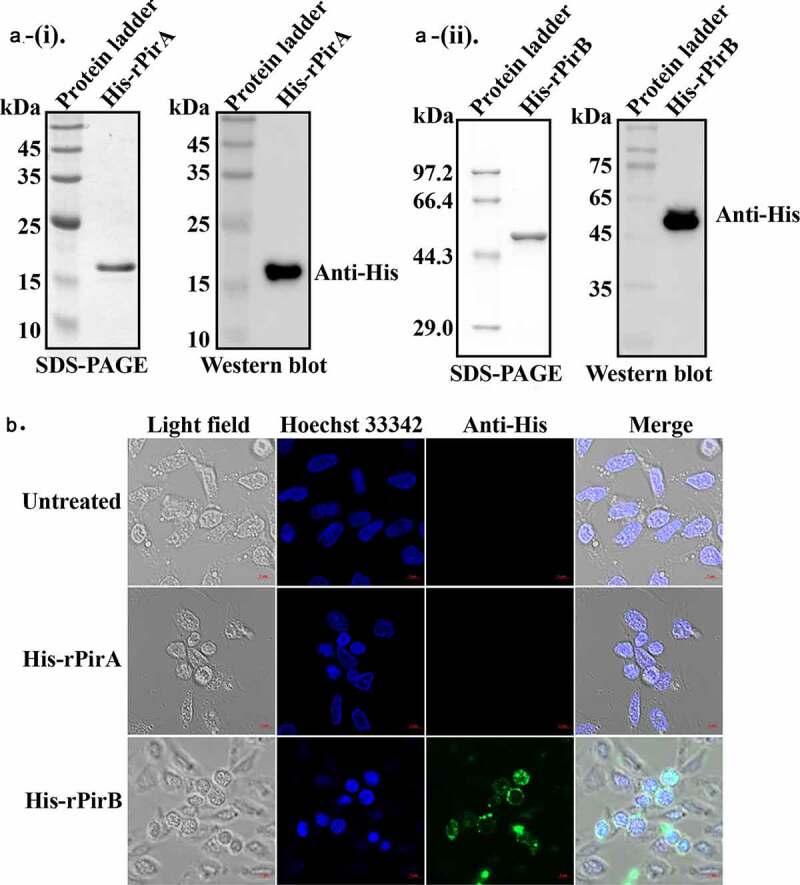
The PirB toxin protein of VP_AHPND_ can localize to shrimp hemocytes. (a) SDS-PAGE and Western blot analysis of recombinant His-rPirA and His-rPirB proteins, (i) His-rPirA, (ii) His-rPirB. (b) Localization of Pir toxin proteins in primary hemocytes of *P. vannamei* was analyzed by confocal microscopy. Hemocytes treated with 0.1 µM recombinant His-rPirA or His-rPirB proteins for 2 h, followed by immunofluorescence analysis using an anti-His antibody. The images shown represent one of three independent experiments

The PirA and PirB toxin protein of VP_AHPND_ were cloned, expressed, and the purified recombinant proteins (His-rPirA and His-rPirB) determined by SDS-PAGE and Western blot analysis (Figure 1a). After ascertaining that the correct were obtained (expected molecular weights of about 15 and 50 kDa, respectively), primary shrimp hemocytes were treated with His-rPirA and His-rPirB, followed by immunofluorescence analysis to determine their cellular localization. Immunofluorescence signals of only His-rPirB were found mainly in the cytoplasm and nucleus of treated cells but not in His-rPirA or untreated cells (Figure 1b), which suggest that only PirB protein could enter shrimp hemocytes.

### PirB protein interacts with histones in shrimp hemocytes

**Figure 2.**
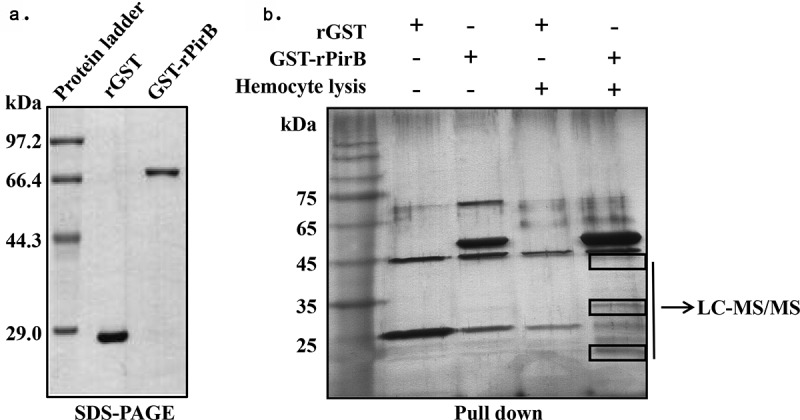
Analysis of protein-protein interaction between PirB toxin protein of VP_AHPND_ and shrimp hemocytes proteins. (a) SDS-PAGE analysis of recombinant rGST and GST-rPirB proteins. (b) pull-down analysis of the interaction between GST-rPirB and proteins in hemocytes lysate

Recombinant GST-tagged PirB proteins (rGST and GST-rPirB) were cloned, expressed (with IPTG induction), and purified, after which the proteins were ascertained by SDS-PAGE analysis. Single bands corresponding to 26 kDa and 75 kDa, representing rGST and GST-rPirB, respectively, were found on the gels (Figure 2a). Next, the puriﬁed rGST and GST-rPirB proteins were used for pull-down analysis to determine their interaction with shrimp hemocyte lysate proteins. As shown in Figure 2b, three diﬀerential bands with approximate sizes 40–45 kDa, 30–35 kDa, and 10–20 kDa were observed in the GST-rPirB + hemocyte lysate samples compared with control (rGST + hemocytes lysate). The three bands were excised and subjected to LC-MS/MS analysis, followed by a search of the generated LC-MS/MS spectra against the NCBI database (http://www.ncbi.nlm.nih.gov/) using MASCOT (V2.3.02). The search identified 20 putative PirB interacting proteins (Supplementary Table S1). Because the excised protein bands used for the LC-MS/MS analysis had molecular masses in the ranges: 40–45 kDa, 30–35 kDa, and 10–20 kDa, respectively, all proteins within this range (i.e. 10–45 kDa) were selected for further analysis. Based on this criterion, 13 proteins were selected (Table 2) including histone H4, which had the highest protein score, and histone H3.

**Table 2.** Summary annotated proteins identified to interact with PirB

No.	Description	CoverPercent	MW	PepCount	Peptide	Score
1	histone H4-like	17.09%	12,779.8	2	R.DAVTYTEHAK.R/R.ISGLIYEETR.G	54.62
2	hypothetical protein	5.15%	25,231.21	2	K.LPEDLR.Q/R.ETEICR.	52.58
3	beta-I tubulin	11.84%	16,542.35	1	K.GHYTEGAELVDSVLDVVR.K	47.52
4	beta-actin	4.19%	18,651.25	1	K.IIAPPER.K	42.51
5	Histone H3	5.60%	14,004.13	1	R.STELLIR.K	26.85
6	hypothetical protein	2.89%	41,523.53	1	R.QMPEVASGEDK.S	23.81
7	tropomyosin	17.50%	32,849.11	1	R.SLSDEER.M	23.26
8	growth arrest-specific protein 2-like	6.02%	15,369.19	1	R.LAEVISRR.A	22.53
9	chloride channel CLIC-like protein 1-like	2.15%	32,339.6	1	K.VAESRR.H	21.6
10	minus strand Zinc finger protein on ecdysone puffs	2.06%	32,685.56	1	R.GSLQVR.T	21.27
11	uncharacterized protein	2.42%	25,549.13	1	R.RPSLPR.A	20.96
12	minus strand hypothetical protein	4.55%	27,658.57	1	K.GVMQEAVAMLR.E	20.64
13	tropomyosin-2 isoform 4	3.97%	29,050.18	1	R.IQLLEEDLER.S	20.63

**Figure 3.**
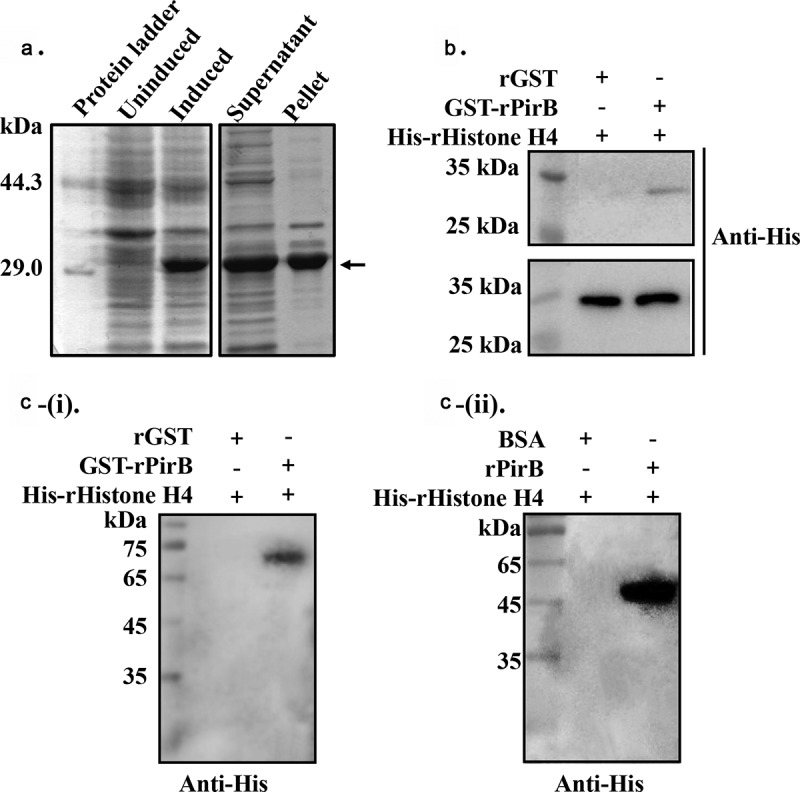
The PirB toxin protein interacts with histone H4. (a) SDS-PAGE analysis of the expression of His-rhistone H4. (b) GST pull-down analysis of the interaction between GST-rPirB and His-rhistone H4. (c) The PirB toxin protein interacts with histone H4, (i) Far-Western blot analysis of the interaction between GST-rPirB and His-rhistone (rGST as negative control). (ii) Far-Western blot analysis of the interaction between rPirB and His-rhistone (BSA as negative control)

**Figure 4.**
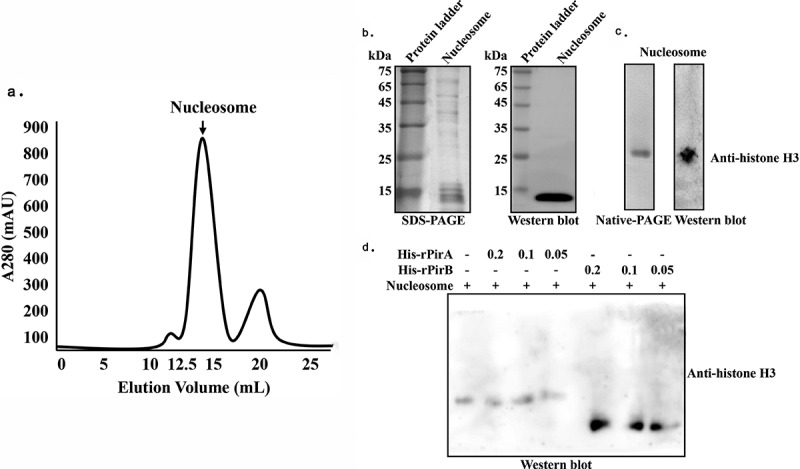
The interaction between PirB toxin protein and histones. (a) Purification of nucleosome proteins by gel ﬁltration chromatography. (b) SDS-PAGE, Native-PAGE and Western blot analysis of purified nucleosomes. (c) Native-PAGE and Western blot analysis of the interaction between His-rPirB and nucleosome proteins. Purified nucleosomes treated with different concentrations (0.2, 0.1, and 0.05 µM) of recombinant His-rPirA or His-rPirB proteins and analyzed by Native-PAGE and Western blot using anti-histone H3 antibody

To examine the interaction between PirB and histone H4 (*Pv*histone H4) in shrimp hemocytes, His-tagged *Pv*histone H4 was cloned, expressed, and the recombinant protein (His-rhistone H4) analyzed by SDS-PAGE. A single protein band (molecular weight ~30 kDa) was observed (Figure 3a). Next, GST pull-down and Far-Western blot analysis were applied to determine the interaction between GST-rPirB and His-rhistone H4. As shown in Figure 3b, the GST pull-down results revealed that His-rhistone H4 could interact with GST-rPirB, but not rGST. Similarly, the Far-Western blot results showed that His-rhistone H4 could bind to GST-rPirB, but not rGST (Figure 3c (i)), with His-rhistone H4 also capable of binding to rPirB, but not BSA (Figure 3c (ii)). In view of the fact that the mass spectrometry results revealed a possible interaction of PirB with histone H4 and histone H3, we speculated that PirB could bind to histone proteins. To further confirm the interaction between PirB and histones, *Drosophila* S2 cells nucleosome proteins (due to unavailability of shrimp cell lines) were purified using molecular sieve gel chromatography and ascertained by SDS-PAGE, Native-PAGE and Western blot analysis. A single nucleosome protein was obtained (Figure 4a-C), which indicates that the purified product could be used for subsequent experiments. Next, the purified nucleosomes were treated with His-rPirA or His-rPirB, before being resolved on Native-PAGE and then blotted onto PVDF membranes, followed by Western blot analysis using anti-histone H3 antibody. As shown in Figure 4d, the purified nucleosomes could bind with His-rPirB, but not His-rPirA, in a concentration-dependent manner. When the nucleosome proteins were treated with His-rPirB, their interaction caused a change in the position of nucleosome proteins on Native-PAGE, with no change observed in His-rPirA treated or untreated groups. These results demonstrate that PirB could specifically bind to histones.

### *The PirB protein of* Vibrio parahaemolyticus *induces hemocytes apoptosis*

**Figure 5.**
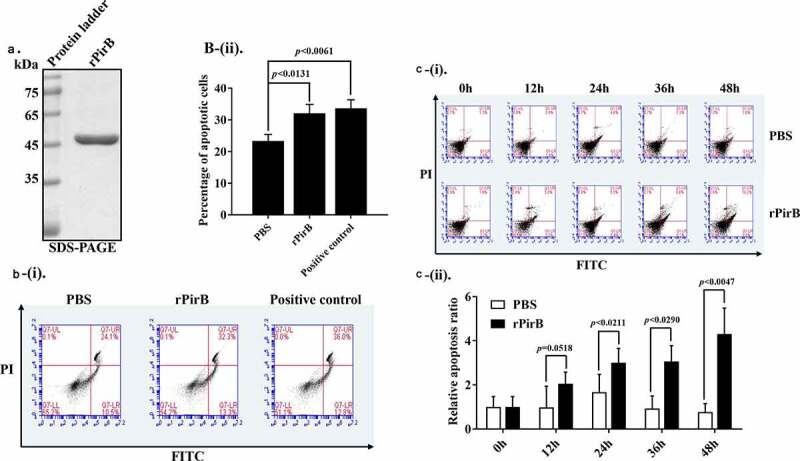
The PirB protein induces hemocytes apoptosis. (a) SDS-PAGE analysis of recombinant rPirB proteins. (b) Apoptosis of primary hemocytes after *in vitro* treatment with rPirB protein. The number of apoptotic cells (hemocytes) was determined at 4 h post-treatment with rPirB (0.1 µM), PBS (negative control) or Apoptosis inducer A (positive control) using by Annexin V-FITC/PI double staining. The Q3-LR region of the graph represents early apoptotic cells, while the Q3-UR region represents late apoptotic cells. (i) Number of apoptotic cells. (ii) Percentage of apoptotic of hemocytes post-treatment with rPirB. (c) Analysis of shrimp hemocytes apoptosis after *in vivo* treatment with rPirB. Hemocytes were collected at 0, 12, 24, 36, and 48 h from shrimp injected with 0.8 µM rPirB proteins or PBS (negative control) and then analyzed by flow cytometry. (i) Number of apoptotic cells. (ii) Relative apoptosis ratio of hemocytes. The relative apoptosis ratio for the control at each time point was set to 1. Data represent mean ± SD (n = 4) for three independent experiments. Statistical significance was determined using Student’s t-test relative to PBS treatment and the *p*-values indicated on plots

After establishing that PirB could interact with histones, we explored the functional significance of this interaction. First, the effects of purified endotoxin-free rPirB protein (Figure 5a) on primary shrimp hemocytes were examined. Hemocyte apoptosis was determined by flow cytometry after treating primary shrimp hemocytes with purified endotoxin-free rPirB protein, PBS (negative control), or apoptosis inducer A (positive control) for 4 h, followed by Annexin V-FITC/PI staining. As shown in Figure 5b, a significantly higher percentage of apoptotic cells was observed in the rPirB-treated cells than in control (PBS). The *in vivo* effect of PirB on shrimp hemocytes was also determined by injecting shrimp with purified endotoxin-free rPirB protein or with sterile PBS as negative control, followed by harvesting hemocytes from hemolymph, Annexin V-FITC/PI staining, and flow cytometry analysis. It was observed that the proportion of apoptotic cells in rPirB-injected shrimp were significantly higher at 24 h (*p <* 0.05), 36 h (*p <* 0.05) and 48 h (*p < *0.01) compared with PBS injected shrimp at the same time points (Figure 5c). These results suggest that PirB protein from VP_AHPND_ induces apoptosis in shrimp hemocytes under both *in vitro* and *in vivo* conditions.

### The dephosphorylation of histone H3 induces apoptosis in hemocytes

**Figure 6.**
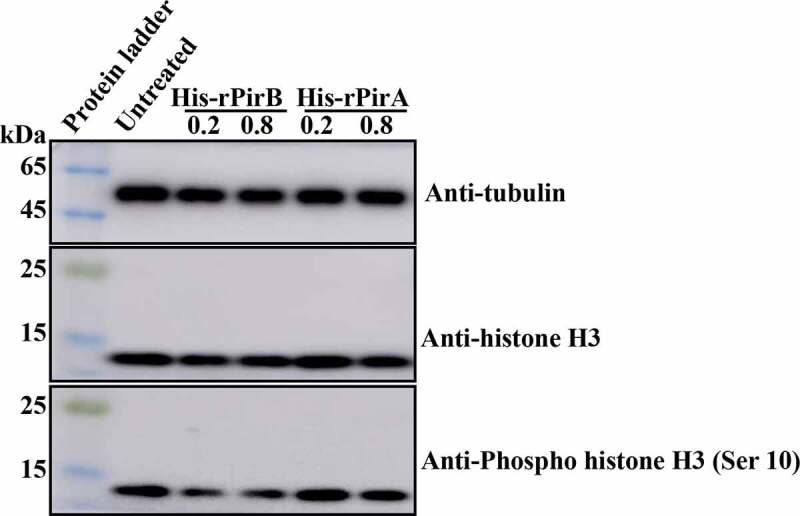
The PirB toxin protein induces dephosphorylation of histone H3 on Serine 10. Primary hemocytes were treated with His-rPirB or His-rPirA for 2 h, followed by Western blot analysis to detect total tubulin, histone H3, and phosphorylation levels of histone H3 on Serine 10

Given that bacterial toxins induce histone H3 dephosphorylation on Serine 10 during early infection and apoptosis [28,29], we examined whether PirB from VP_AHPND_ could also cause the dephosphorylation of histone H3 (Ser 10). Thus, primary hemocytes were treated with endotoxin-free His-rPirB or His-rPirA proteins for 2 h before being lysed and the phosphorylation of histone H3 (Ser10) analyzed by Western blot. As shown in Figure 6, in the His-rPirB-treated samples, histone H3 (Ser10) was dephosphorylated compared with control (untreated and His-rPirA treated). The dephosphorylation was, however, not dependent on the concentration of PirB. These results suggest that the apoptosis induced by PirB protein in hemocytes could result from the dephosphorylation of histone H3 (Ser 10) through interaction between PirB protein and histones.

## Discussion

Acute hepatopancreatic necrosis disease (AHPND) has had devastating effects on shrimp culture in the last couple of years [8]. Despite the extensive research work that has sought to understand the causative agent (s) and factors that promote AHPND, the disease’s pathogenesis and the molecular mechanisms involved are still not well understood [14]. Recently, it has been reported that the AHPND strain of *V. parahaemolyticus* does affect not only the gastrointestinal system of shrimp (i.e. hepatopancreas, stomach, etc.) but also hemocytes to dysregulate the expression of apoptosis-related genes [18]. Therefore, the current study sought to explore the effect of AHPND on shrimp hemocytes, especially the effects and potential mechanism of action of the Pir toxin proteins on hemocyte apoptosis.

Although PirA and PirB toxin proteins are regarded as the key pathogenic factors in AHPND [11], when shrimp hemocytes were treated with recombinant PirA and PirB proteins (His-rPirB and His-rPirA), only His-rPirB was found to enter and detected within cells (Figure 1b). Thus, the pathogenic damage induced by VP_AHPND_ through its secreted toxin proteins is not limited to the gastrointestinal system but extends to other tissues/organs such as hemocytes. This observation is synonymous with the study by Salles *et al*., where the Anthrax toxin protective antigen (PA) of *Bacillus anthracis* was shown to bind to an anthrax toxin receptor 1,2 (ANTXR1,2), cell surface receptors of macrophages, to form heptameric pores, to allow it translocate lethal factors (LF) or edema factors (OF) into macrophages [30]. The PirB toxin is reported to first enter the stomach before being transferred to the hepatopancreas to cause cell sloughing and cell death [14,31]. Interestingly, our current data shows that the PirB toxin could be found in hemocytes, which suggest that it might have been transported there or to other tissues from the gastrointestinal system via hemolymph.

Nucleosomes comprise of DNA complexed with histones H1, H2A, H2B, H3 and H4 [32]. The PirB toxin protein could interact with histone H4 in shrimp hemocytes (Figs. 2 and 3). Mass spectrometry analysis also identified histone H3 as a putative PirB interacting protein (Table 2), while PirB was found to specifically interact with histones isolated from Drosophila S2 cells (Figure 4). These results thus confirm that PirB proteins can enter hemocytes to interact with nucleosome proteins. As essential components of nucleosomes, histones are targets of bacterial or viral toxins. The interaction between toxins and histones could disrupt nucleosome assembly, thereby affecting the modification of nucleosomes and repair of host DNA, which eventually leads to apoptosis [33]. For instance, the nonstructural protein ICP11 of WSSV binds with histone H2A and H3 to prevent DNA binding, hence destroying the assembly of nucleosomes [34]. Similarly, the 5-methylcytosine-specific DNA methyltransferase, Rv2966c, secreted by *Mycobacterium tuberculosis* can bind to histone H3 and H4 to affect their methylation and therefore assist in invading host cells [35]. The ability of PirB to interact with histones, as observed in this study, suggests that upon entry into shrimp hemocytes, PirB binds to histones to induce apoptosis. Under both *in vitro* and *in vivo* conditions, rPirB was found to induce hemocyte apoptosis (Figure 5), synonymous with the studies reported by Nimsanor et al. [36].

In addition to methylation, phosphorylation and acetylation of histones also play essential roles in pathogenic bacteria’s ability to invade host cells to cause apoptosis [28,37,38]. The phosphorylation of histone H3 (Ser 10) is the most common histone modification induced by pathogens. It has been shown that *Streptococcus pneumoniae* infection promotes histone H3 (Ser 10) dephosphorylation [39], which has also been observed after treatment of epithelial cells with purified bacterial toxins such as PLY of *S. pneumoniae* [28]. In the current study, the treatment of shrimp hemocytes with PirB also resulted in the dephosphorylation of histone H3 (Ser 10) (Figure 6). We believe that when PirB enters hemocytes, it interacts with histones, thereby affecting the phosphorylation of histone H3 (Ser 10) and inducing apoptosis. In spite of this, the mechanisms involved when changes in histone H3 (Ser 10) phosphorylation triggers apoptosis-related pathways that affect nucleosome assembly, and the resultant nuclear damage or other biological response remains an open question.

**Figure 7.**
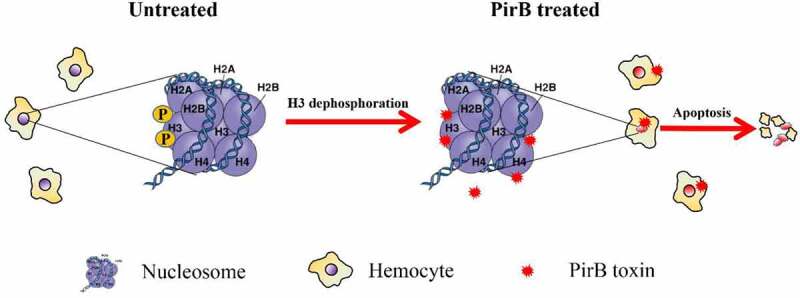
A proposed model shows that PirB induces apoptosis in shrimp hemocytes. When primary hemocytes of *Penaeus vannamei* are treated with the Pir proteins from Vibrio parahaemolyticus, the PirB protein enters the cytoplasm and nuclei. Once in the nucleus, the PirB protein interacts with histone proteins to dephosphorylate histone H3 and therefore induce apoptosis in hemocytes

Most studies have revealed that the AHPND causing bacteria usually release their toxins after invading the digestive tract, which results in the production of the characteristic symptoms [14,17,40]. Although the details of the molecular mechanisms involved in the pathogenesis of AHPND are still unknown, it is believed that PirA and PirB form a heterodimer that binds to receptors, after which PirB enters the cell through pore formation and then circulates to the hepatopancreas where it exerts cellular damage including sloughing of epithelial cells into the lumen (see recent reviews by [41,42]). Thus, damage to the hepatopancreatic tubules allows PirB toxin to be released into the intertubular space, which is then carried into hemolymph to infect hemocytes. Once it enters hemocytes, PirB could then bind to histones in nucleosomes to cause the dephosphorylation of histone H3 and to induce apoptosis (Figure 7). Given that PirB was localized to the cytoplasm, it suggests that PirB also interacts with other cytosolic proteins to induce other functions; hence, the mechanism of AHPND pathogenesis could be a complicated process that requires additional studies to delineate.

In summary, the current study reveals for the first time that the PirB toxin protein of VP_AHPND_ induces hemocyte apoptosis by binding to histones in nucleosomes. While these are interesting findings, the specific mechanisms involved would have to be further explored using nucleosome disassembly assay, histone modifications, etc. These results provide useful information for understanding the molecular pathogenesis of AHPND in crustaceans.

## Supplementary Material

Supplemental Material
