# Supplementary material for: The PirB toxin protein from Vibrio parahaemolyticus induces apoptosis in hemocytes of Penaeus vannamei: PirB induces shrimp hemocytes apoptosis
Source: Virulence. 2021 Jan 25;12(1):481–92. doi: 10.1080/21505594.2021.1872171 (PMC7834086; doi:10.1080/21505594.2021.1872171)
Supplement: Supplemental Material [file KVIR_A_1872171_SM8567.doc]

**Table S1. Summary of identified annotated proteins by LC-MS/MS**

| No. | Description | CoverPercent | MW | PepCount | Peptide | Score |
| --- | --- | --- | --- | --- | --- | --- |
| 1 | histone H4-like | 17.09% | 12779.8 | 2 | R.DAVTYTEHAK.R/R.ISGLIYEETR.G | 54.62 |
| 2 | hypothetical protein | 5.15% | 25231.21 | 2 | K.LPEDLR.Q/R.ETEICR. | 52.58 |
| 3 | beta-I tubulin | 11.84% | 16542.35 | 1 | K.GHYTEGAELVDSVLDVVR.K | 47.52 |
| 4 | beta-actin | 4.19% | 18651.25 | 1 | K.IIAPPER.K | 42.51 |
| 5 | similar to G protein pathway suppressor 1 | 1.19% | 55569.01 | 1 | K.LDTDLK.N | 34.98 |
| 6 | Histone H3 | 5.60% | 14004.13 | 1 | R.STELLIR.K | 26.85 |
| 7 | myosin-IXb isoform 3 | 2.07% | 71375.03 | 1 | R.SGQGASEASSLPR.T | 26.72 |
| 8 | JHE-like carboxylesterase 1 | 1.61% | 62384.5 | 1 | K.RDEDLFVSR.I | 25.44 |
| 9 | hypothetical protein TTHERM_00437740 | 0.77% | 103530.3 | 1 | K.EAMEIIR.K | 24.08 |
| 10 | hypothetical protein CAPTEDRAFT_221097 | 2.89% | 41523.53 | 1 | R.QMPEVASGEDK.S | 23.81 |
| 11 | splicing factor 3B subunit 1-like | 0.52% | 129365.8 | 1 | R.IVDDLK.D | 23.32 |
| 12 | tropomyosin | 17.50% | 32849.11 | 1 | R.SLSDEER.M | 23.26 |
| 13 | RNA polymerase II associated protein 1 | 0.89% | 75573.35 | 1 | K.LVQTPR.L | 22.59 |
| 14 | growth arrest-specific protein 2-like | 6.02% | 15369.19 | 1 | R.LAEVISRR.A | 22.53 |
| 15 | chloride channel CLIC-like protein 1-like | 2.15% | 32339.6 | 1 | K.VAESRR.H | 21.6 |
| 16 | minus strand Zinc finger protein on ecdysone puffs | 2.06% | 32685.56 | 1 | R.GSLQVR.T | 21.27 |
| 17 | uncharacterized protein LOC608818 | 2.42% | 25549.13 | 1 | R.RPSLPR.A | 20.96 |
| 18 | minus strand hypothetical protein CAPTEDRAFT_229048 | 4.55% | 27658.57 | 1 | K.GVMQEAVAMLR.E | 20.64 |
| 19 | tropomyosin-2 isoform 4 | 3.97% | 29050.18 | 1 | R.IQLLEEDLER.S | 20.63 |
| 20 | E3 ubiquitin-protein ligase HECTD1-like | 0.46% | 215244.2 | 1 | K.SQHMVVGAR.V | 20.26 |
